# Estimating Ross 308 Broiler Chicken Weight Through Integration of Random Forest Model and Metaheuristic Algorithms

**DOI:** 10.3390/ani14213082

**Published:** 2024-10-25

**Authors:** Erdem Küçüktopçu, Bilal Cemek, Didem Yıldırım

**Affiliations:** Department of Agricultural Structures and Irrigation, Ondokuz Mayıs University, Samsun 55139, Türkiye; bcemek@omu.edu.tr (B.C.); ydidem19@gmail.com (D.Y.)

**Keywords:** weight, random forest, poultry, optimization, hyperparameter

## Abstract

For accurate estimation of broiler chicken weight (CW), a novel hybrid method was developed in this study where several benchmark methods, including Particle Swarm Optimization (PSO), Genetic Algorithm (GA), Ant Colony Optimization (ACO), Differential Evolution (DE), and Gravity Search Algorithm (GSA), were employed to adjust the Random Forest (RF) hyperparameters. The performance of the RF models was compared with that of classic linear regression (LR). With this aim, data (temperature, relative humidity, feed consumption, and CW) were collected from six poultry farms in Samsun, Türkiye, covering both the summer and winter seasons between 2014 and 2021. The results demonstrated that PSO and ACO significantly enhanced the performance of the standard RF model in all periods. Specifically, the RF-PSO model achieved a significant improvement by reducing the Mean Absolute Error *(MAE)* by 5.081% to 60.707%, highlighting its superior prediction accuracy and efficiency. The RF-ACO model also showed remarkable *MAE* reductions, ranging from 3.066% to 43.399%, depending on the input combinations used. In addition, the computational time required to train the RF models with PSO and ACO was considerably low, indicating their computational efficiency. These improvements emphasize the effectiveness of the PSO and ACO algorithms in achieving more accurate predictions of CW.

## Simple Summary

The rapid development of computer technology in recent years has led to innovative solutions in various fields, including poultry farming, where weight is an important physical growth indicator used to evaluate efficiency by comparing it with the amount of feed consumed. This paper presents a novel hybrid approach that combines a Random Forest model with metaheuristic algorithms to estimate the weight of chickens. The results of the study show that these hybrid models are very promising in terms of making accurate and efficient predictions about the weight of chickens. This advancement sets the stage for more effective weight management in poultry farming, ultimately leading to better resource utilization and improved productivity.

## 1. Introduction

Global meat consumption is projected to increase in the future, driven by the continuously growing world population and increasing demand for animal food proteins [1]. The poultry industry is experiencing rapid growth, particularly in developing countries, driven by factors such as urbanization, population growth and rising incomes. Over the years, white meat has become more popular worldwide, increasing the consumption of poultry products, with broiler meat being the most sought-after meat [2]. In 2019, poultry was the most consumed meat worldwide, with an average consumption of 14.7 kg per capita. It was followed by pork at 11.1 kg per capita, beef at 6.4 kg per capita, and sheep and goat meat at 1.8 kg per capita [3]. This increase in demand is due to poultry meat being a good source of protein that is high-quality and affordable compared to other meats [4].

In broiler production, weight is a key physical growth indicator for assessing efficiency as it is compared with the amount of feed consumed [5]. Knowing the average weight and weight range at slaughter in advance is essential for flock managers [6]. An accurate estimate of the flock weight distribution and average weight helps in the selection and slaughter of broilers. The traditional method of measuring weight is the manual method, in which the chicken is caught and weighed with an electronic scale [7]. The process increases labor costs, endangers chicken welfare, affects quality and yield, and can even lead to chicken death [8]. In addition, this measurement is laborious and stressful for the animals [9].

In modern broiler houses, automatic weighing systems are designed so that the birds are weighed when they voluntarily visit the weighing platform [10,11]. These systems can provide real-time weight estimates without physically handling the birds, reducing stress and improving welfare. By minimizing human intervention, they also reduce labor costs and improve the accuracy of weight assessments. A significant disadvantage of these automatic platform systems is that individual birds, especially heavier ones, may visit the platform less frequently, making it difficult to accurately measure their weight [12]. To address this challenge, researchers have developed different models for estimating weight [13,14,15]. These models, however, often depend on empirical formulas and linear regressions which cannot fully account for the complex and non-linear interactions affecting bird weight. There is, therefore, a need for alternative methods (e.g., machine learning (ML) algorithms) to improve the accuracy and reliability of broiler weight estimation.

In recent decades, neural network (NN) models have become a significant focus in poultry production research. The NN models provide advanced predictive capabilities by analyzing large data sets of poultry growth factors such as feed intake, environmental conditions, and health indicators. Unlike traditional methods, NNs can automatically learn complex relationships between these factors and weight, resulting in more accurate and efficient predictions. This technology minimizes human error and reduces labor-intensive manual data collection. Several studies in the literature have estimated broiler weight using NN. For instance, in [16], a three-layer feedforward NN was implemented to estimate the body weight of broiler chickens. In [17], the performance of broilers was predicted using an NN. In the study of [18], NN models were trained and compared with actual growth data to predict the growth of broilers over the next 50 days. In [19], a differential recurrent NN was proposed for chicken growth modeling. Meanwhile, the authors of [20] investigated the feasibility of a radial basis function NN for chicken growth analysis. The study in [21] developed a NN model that linked feed intake, weight gain, and feed conversion. In [22], dynamic NN prediction models were trained on data from 12 batches of broilers that considered environmental and behavioral factors to predict future weights and showed successful predictions of growth patterns. The study in [23] showed that their NN model successfully estimated feed conversion under heat stress with satisfactory results.

The authors of [24] identified Random Forest (RF) as the most interpretable non-parametric ML algorithm. Additionally, the RF technique is noted for its ability to effectively capture non-linear dependencies between predictors and dependent variables [25]. Moreover, numerous studies have confirmed the superior predictive performance of RF [26,27,28].

Hyperparameter optimization is essential for improving the prediction accuracy of ML models [29]. Numerous studies have shown that metaheuristic-enhanced ML models perform better than standalone ML models [30,31,32], which tend to omit valuable information when used in isolation. Therefore, in recent years, researchers have increasingly turned to evolutionary algorithms to enhance model performance during training.

To our knowledge, RF and hybrid RF-based metaheuristic models for broiler weight prediction have not yet been developed. Considering the advantages of RF and the research gap in its application for broiler weight prediction, this research investigates the potential of using hybrid RF-based models for this purpose.

The novelty of this research lies in the first-time development of hybrid RF-based metaheuristic algorithms specifically designed for broiler weight prediction. To achieve this, we employed five powerful metaheuristic optimization algorithms (Particle Swarm Optimization (PSO), Genetic Algorithm (GA), Ant Colony Optimization (ACO), Differential Evolution (DE), and Gravity Search Algorithm (GSA)) to determine the optimal hyperparameters of the RF model to predict broiler weights. In selecting the metaheuristic algorithms, we chose PSO, GA, ACO, DE, and the GSA because of their ability to solve non-linear high-dimensional problems and find optimal solutions. These algorithms were chosen for their computational efficiency and low cost [33,34,35], which makes them ideal optimizers for the RF model in this study.

The main contributions of this study are summarized as follows:

- The linear regression (LR) and RF models were developed to predict the weight of ROSS 308 broiler chickens.
- A novel approach that combines RF with five powerful metaheuristic optimization algorithms (PSO, GA, ACO, DE, and the GSA) was created to enhance weight prediction.
- The accuracy, applicability, and reliability of these algorithms were compared using data collected weekly from six poultry farms in Samsun, Türkiye, during the summer and winter seasons of 2014–2021.

The rest of this article is organized as follows. Section 2 details the LR, RF, and metaheuristic optimization algorithms (PSO, GA, ACO, DE, and the GSA) that were used to estimate broiler weight. Section 3 outlines the methodology employed, including the experimental setup, data set, data pre-processing, and performance criteria for the models. Section 4 compares the predictive performance of the models, presents numerical results, and discusses the study’s findings. Finally, Section 5 summarizes the conclusions and offers recommendations for future work.

## 2. Background

### 2.1. Random Forest (RF)

RF is a flexible, non-parametric ensemble learning technique first developed by Breiman [36]. It combines elements of decision trees and regression and is a powerful method that has gained widespread popularity in ML due to its robustness and versatility. RF is effective for both classification and regression tasks and is known for its ability to resist overfitting [37]. By aggregating the predictions of multiple decision trees trained on different subsets of the data, RF reduces variance and improves generalization performance.

The RF offers several notable advantages that increase its usefulness in various applications. One main advantage is its ability to handle non-linear relationships between features and the target variable, making it suitable for complex data sets. Moreover, RF is robust in terms of overfitting due to its ensemble nature, which combines the predictions of multiple decision trees, so it performs well even in large data sets with high dimensionality. The model also provides valuable insights into the importance of features, allowing practitioners to identify and prioritize the most influential variables in their analysis. Additionally, the RF is versatile for both classification and regression tasks, and it is insensitive to noise in the data, so accuracy is maintained even with irrelevant or misleading features. Collectively, these advantages position RF as a powerful and flexible tool within the field of ML. However, like any method, RF has its limitations. The biggest disadvantage is its lack of interpretability compared to simpler models such as decision trees. In addition, training an RF model can be very computationally intensive, especially with large data sets and a high number of trees [38]. Despite these limitations, RF remains the first choice for many ML tasks due to its robust performance, scalability, and ease of use [39,40,41].

### 2.2. Metaheuristic Algorithms

Optimization and computational intelligence play a key role in various disciplines concerned with maximizing the use of resources. Their importance is both current and future-oriented. At the heart of optimization are the mathematical models and solution techniques used to solve problems. As these problems become larger and more complex, conventional optimization methods often prove inadequate. Therefore, researchers have turned to ML-based, nature-inspired algorithms known as metaheuristics. These algorithms, which are based on simple rules, can effectively solve large, complicated real-world problems.

Metaheuristics include different types of algorithms, including evolutionary, bio-inspired, physics-based, socio-inspired, and swarm-based algorithms [42]. In this study, the hyperparameters of the RF were optimized using different algorithms: GA and DE, from the category of evolutionary algorithms; the GSA, from the category of physics-based algorithms; and PSO and ACO, from the category of swarm-based algorithms.

- Genetic Algorithm (GA)

GAs are advanced search techniques inspired by natural evolutionary processes. They mimic the mechanisms of natural selection, population genetics, and evolution, including the persistence of the fittest individuals, maintenance of genetic diversity, inheritance, and occasional mutation [43]. GAs are particularly effective for tackling combinatorial optimization problems that are difficult to solve with traditional analytical methods.

As a Darwinian evolutionary model based on stochastic methods, GAs differ from conventional optimization techniques by exploring multiple potential solutions simultaneously rather than following a single path. The GA process involves two main phases: evaluation and generation. In each iteration, a population of candidate solutions is evaluated based on a fitness function. Each solution is represented by a chromosomal chain where each gene can have various values [44]. The generation phase evolves the population through genetic operators such as selection, crossover, and mutation. In this study, a crossover rate of 0.01 and a mutation rate of 0.7 were applied. Detailed information about GA can be obtained from Kramer and Kramer [45].

- Differential Evolution (DE)

DE, introduced by Storn and Price [46] in 1997, is a straightforward yet powerful algorithm for global optimization that is particularly effective in multimodal scenarios. Originally developed for the optimization of functions with continuous and discrete numerical variables, DE has also shown promise in solving combinatorial problems.

DE is a type of evolutionary algorithm (EA), which is a category of population-based methods that use mutation, recombination and selection to evolve candidate solutions towards optimality. Unlike some other EAs that mimic certain natural processes—such as ant colonies, bee swarms, the immune system, or social interactions—DE is based on a unique approach. It uses a population-based strategy in which mutation is driven by differences between randomly selected individuals, a method that offers significant advantages in terms of optimization.

- Gravitational Search Algorithm (GSA)

The GSA is a population-based, iterative metaheuristic algorithm designed for solving continuous optimization problems. It simulates Newton’s law of gravity and the laws of motion by applying these principles to a population of objects in a continuous space [47].

In the GSA, the search space is analogous to the universe, where all objects experience gravitational forces based on their masses and distances from one another. The algorithm has been significantly enhanced, with adaptations being made for binary, discrete, continuous, single-objective, and multi-objective optimization problems. The variations in these versions are reflected in the design variables and objective functions employed. The GSA has proven itself in a variety of complex optimization problems, especially in the field of engineering. Detailed information about the theory and application of the GSA can be obtained from Hashemi, Dowlatshahi [48].

- Particle Swarm Optimization (PSO)

Introduced by Kennedy and Eberhart [49] in 1995, the PSO algorithm is a computational method for solving optimization problems. It is based on the social behavior of animals such as birds and insects. PSO uses the process of group communication, where individual members share their discoveries. When a group goes foraging or migrating, those who find the best path first will persuade the others to follow it, even if not all members know it [50].

In PSO, each unit of the population is called a particle and the collective group is referred to as a swarm. Starting from a swarm of particles that is randomly initialized, each particle explores the search space in different directions, tracking its own best positions and those of its neighbors. The particles exchange information about optimal positions and adjust their movements and velocities based on this shared knowledge. This process is iterative, with the particles gradually converging to the best possible solution for the fitness function. For detailed information, please refer to Marini and Walczak [50].

- Ant Colony Optimization (ACO)

The ACO algorithm is a metaheuristic method for solving complex combinatorial optimization problems that was formalized by Dorigo [51]. It is inspired by the natural behavior of real ants that use pheromones to communicate. ACO mimics this process with artificial ants that use pheromone trails to communicate indirectly within a colony. These pheromone trails provide distributed numerical information that guides the ants in creating solutions in a probabilistic manner. As the algorithm progresses, the ants change the pheromone trails according to their search experience to improve solution quality. For a comprehensive overview of ACO, readers should refer to Blum [52].

### 2.3. Linear Regression (LR)

The LR analysis was conducted to determine the linear relation between input variables—temperature (T), relative humidity (RH), and feed consumption (FC)—and the out-put variable, chicken weight (CW) [53].

## 3. Methodology

**Figure 1:**
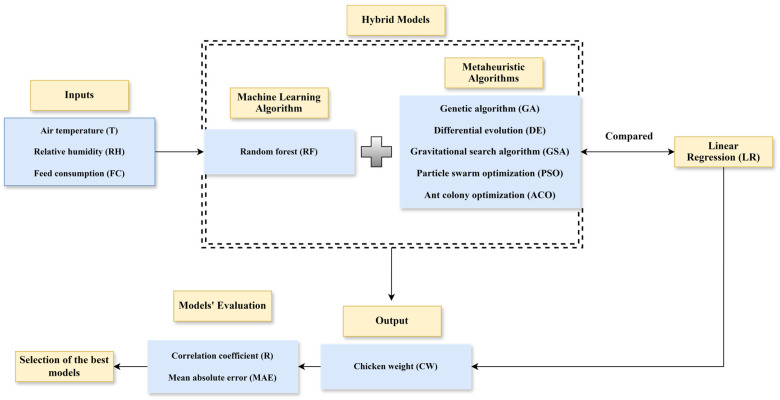
Flowchart of the chicken weight (CW) estimation model.

The hybrid RF models were developed to estimate the CW in poultry houses (Figure 1). Initially, long-term data (2014–2021) (T, RH and FC) from 6 poultry farms were included in this model. Then, CWs were calculated using hybrid RF-based metaheuristic algorithms, and their performance was compared with the LR method. Finally, a statistical analysis was used to determine the best model for estimating CWs.

### 3.1. Experimental Building and Data

The data (T, RH, FC, and CW) for this study were collected from 6 poultry farms in Samsun, Türkiye, weekly in both the summer and winter seasons from 2014 to 2021. The choice to focus on summer and winter seasons allows us to capture the variability in the environmental conditions (T and RH) and their impact on FC and CW. By analyzing data from both seasons, we ensure a more robust data set that accounts for these environmental influences, thereby enhancing the overall comprehensiveness of our analysis. The building dimensions are 90 m in length, 14 m in width, and 4.40 m in height. “Ross 308” chicks, one day old, were reared for a period of 40–42 days. Data were collected weekly during the summer and winter rearing seasons.

### 3.2. Data Pre-Processing

Effective data pre-processing is crucial for improving the performance and accuracy of machine learning models. This process deals with noisy, missing, and inconsistent data. Further pre-processing steps included cleaning, transforming, and splitting the data. To overcome the challenge of different units of measurement, and to mitigate extreme values, the data were standardized to a range of 0–1. To evaluate the models, the data set was first divided into two parts: 80% for training (*n* = 460) and the remaining 20% for testing (*n* = 116). Second, 90% of the training pool was selected as the training set, with the tuning of the hyperparameters on 90% being randomly selected from this training set. These steps were repeated 10 times to ensure the robustness of the evaluation process [54]. The analysis was carried out on a PC running the 64-bit Windows 11 operating system equipped with a Ryzen 7 CPU (AMD, Santa Clara, CA, USA), 3.2 GHz, and 16 GB RAM.

### 3.3. Performance Criteria of Model

To ensure a precise evaluation of the models’ performance in this study, two commonly utilized metrics were applied: the Correlation Coefficient (*R*) and Mean Absolute Error (*MAE*) [53,54]. The *R* metric measures the strength and direction of the linear relationship between the predicted and actual values. A value close to 1 indicates a strong positive correlation, which means that the model predictions closely match the actual values. The *MAE* value quantifies the average absolute difference between the predicted and actual weights. It is a simple measure of prediction accuracy, with lower *MAE* values indicating better model performance.

(1) $$R=\frac{n\left(\sum xy\right)-\left(\sum x\right)\left(\sum y\right)}{\sqrt{\left[n\sum x^{2}-{\left(\sum x\right)}^{2}\right]\left[n\sum y^{2}-{\left(\sum y\right)}^{2}\right]}}$$

(2) $$MAE=\frac{\sum_{i=1}^{n}\left(\left|x-y\right|\right)}{n}$$

where *x* represents the actual value; *y* denotes the predicted value; and *n* is the total amount of data.

## 4. Results and Discussion

Table 1 presents the descriptive statistics for the input variables (T, RH, and FC) and the output variable, (CW) during both the training and testing periods. The statistics include the minimum (Min), maximum (Max), mean (Mean), standard deviation (SD), skewness (Sk), and kurtosis (Kr).

**Figure 2:**
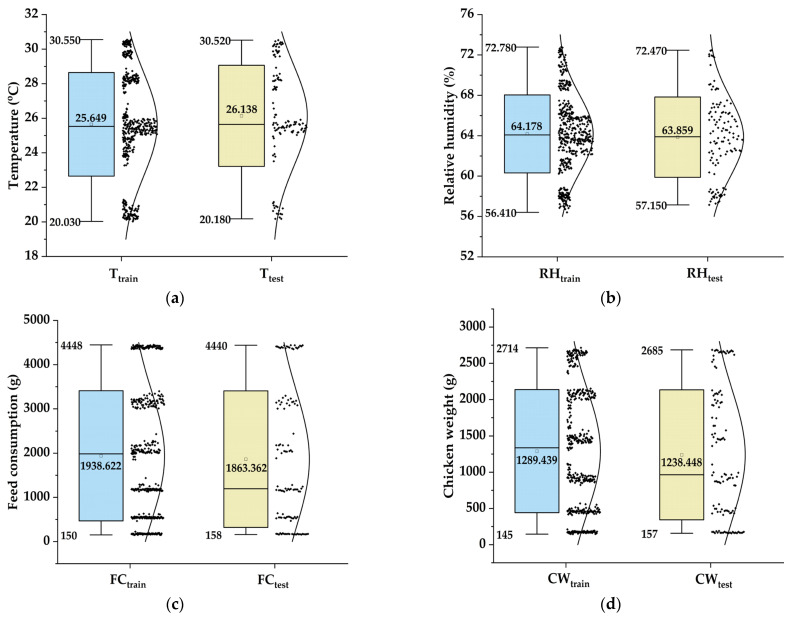
Box plots for (**a**) temperature (T), (**b**) relative humidity (RH), (**c**) feed consumption (FC), and (**d**) chicken weight (CW).

During the training period, the T values exhibited a range from 20.030 °C to 30.550 °C, averaging at 25.649 °C (Figure 2a). The RH values ranged from 56.410% to 72.780%, with an average of 64.178% (Figure 2b). The FC values were between 150 g and 4448 g, with an average of 1938.622 g (Figure 2c). Correspondingly, the CW fluctuated between 145 g and 2714 g, with an average weight of 1289.439 g (Figure 2d).

In the testing period, the T values ranged from 20.180 °C to 30.520 °C, with an average of 26.138 °C (Figure 2a). The RH values varied between 57.150% and 72.470%, averaging at 63.859% (Figure 2b). The FC values ranged from 150 g to 4440 g, with an average consumption of 1863.362 g (Figure 2c). The CW values during this period ranged from 157 g to 2685 g, averaging at 1238.448 g (Figure 2d).

In this study, different combinations of inputs were employed to estimate CW, including (i) T, (ii) RH, (iii) FC, (iv) T and RH, (v) T and FC, (vi) RH and FC, (vii) T, RH and FC.

To investigate the RF model’s capability in accurately predicting CW, this section presents the results of RF models combined with five optimization algorithms: PSO, GA, ACO, DE, and the GSA. It is important to highlight that the testing set was not used during the model development process. The primary objective of using these optimization algorithms was to determine the optimal values for three key hyperparameters: the number of estimators (*n_estimators*), the maximum depth of the trees (*max_depth*), and the minimum number of samples required to split a node (*min_samples_split*). Hyperparameter tuning was performed using a grid search on 90% of a randomly selected training set during each iteration. The tuned hyperparameters for the RF models included *n_estimators* (1:100), *max_depth* (1:50), and *min_samples_split* (1:50).

For all input combination scenarios, Table 2 provides an overview of model performance metrics for models used to predict CW. The performance evaluation of the RF and LR models shows clear differences in predictive ability for different input configurations. The RF model consistently outperformed the LR model, especially when multiple inputs were used. For example, when all three inputs (T, RH, and FC) were combined, the RF model achieved a *MAE* of 23.060 g with an *R* of 0.999 during training and a *MAE* of 38.587 g with an *R* of 0.998 during testing. In contrast, the LR model yielded a *MAE* of 67.876 g with an *R* of 0.995 during training and a *MAE* of 67.019 g with an *R* of 0.995 during testing, highlighting its limitations in capturing the complexity of the data. The ability of the RF model to maintain a low *MAE* and high *R* values across all configurations highlights its robustness and effectiveness in modeling the underlying relationships within the data set and makes it a better choice for CW estimation compared to the LR model.

Before evaluating the accuracy of the RF-based models, a set of control parameter values was tested for each algorithm. The control parameters for the metaheuristic algorithms used in this study (PSO, GA, ACO, DE and the GSA) were determined by the grid search method. For the PSO algorithm, the cognitive component, social component, and inertia weight were varied within the ranges of 0.2 to 2, 1 to 2, and 1 to 2, respectively [55,56]. In the GA, the crossover rate and mutation rate were set between 0.5 and 1 and 0.01 and 0.1, respectively [57]. For the ACO algorithm, the parameters—selection pressure, pheromone evaporation rate, and zeta—were adjusted within the ranges of 0.5 to 1.5, 0.1 to 0.4, and 0.1 to 1, respectively [58,59,60]. For the DE algorithm, the crossover probability and scaling factor were set within 0.1 to 1 and 0.1 to 1 [61,62]. Lastly, in the GSA, the coefficient of decrease and the initial value of the gravitational constant were set in the ranges of 50 to 150 and 10 to 30, respectively [63]. The optimal values were determined for CW estimation (Table 3). Each algorithm used a population size of 30 and performed 200 iterations with 10 runs to ensure robust results.

**Figure 3:**
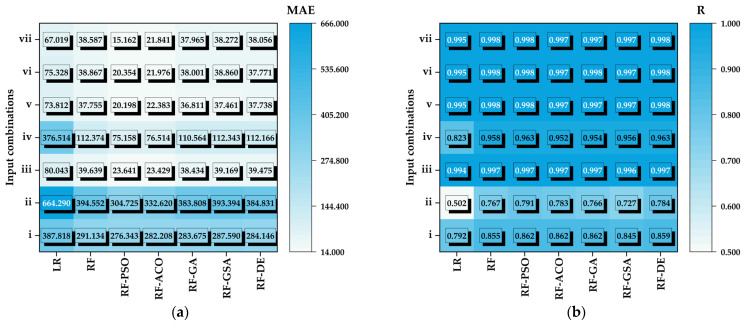
Heatmaps of (**a**) the *MAE* and (**b**) *R* for different inputs and models used in this study for testing data.

Figure 3a,b show heat maps depicting the *MAE* and *R* values for the models used in the study (LR, RF, PSO, ACO, GA, the GSA and DE) for different input combinations. It is evident that the RF-PSO model outperforms the others and has the highest accuracy with the lowest *MAE* and the highest *R* value, making it the most effective and accurate model in this comparison. The ranking of performance, from highest to lowest accuracy, is as follows: RF-PSO > RF-ACO > RF-GA > RF-DE > RF-GSA > RF > LR.

When looking at the input combinations, the most accurate results were obtained with input combination-vii (T, RH, and FC). This combination yielded the lowest *MAE* values and the highest *R* value, indicating that it is the most effective in improving the accuracy of the models compared to the other combinations. It is also evident that, among the inputs, FC is the parameter that influences CW the most. However, in cases where only climatic data are available, the combinations of T and RH can also be used reliably.

The training and testing results of the most promising combinations (vii)—T, RH and FC—of the metaheuristic algorithms are given in Table 4. RF-PSO is characterized by the lowest *MAE* in both training (7.138 g) and testing (15.162 g), together with a high correlation (*R* = 0.999 in training, *R* = 0.998 in testing), indicating a high prediction accuracy. RF-ACO has a higher *MAE* in testing (21.841 g) compared to RF-PSO but still shows a good correlation (*R* = 0.997). The other algorithms (RF-GA, RF-GSA, RF-DE) show comparable performance, with higher error rates in testing, *MAE* values of around 37.965–38.272 g, and *R* = 0.997.

**Figure 4:**
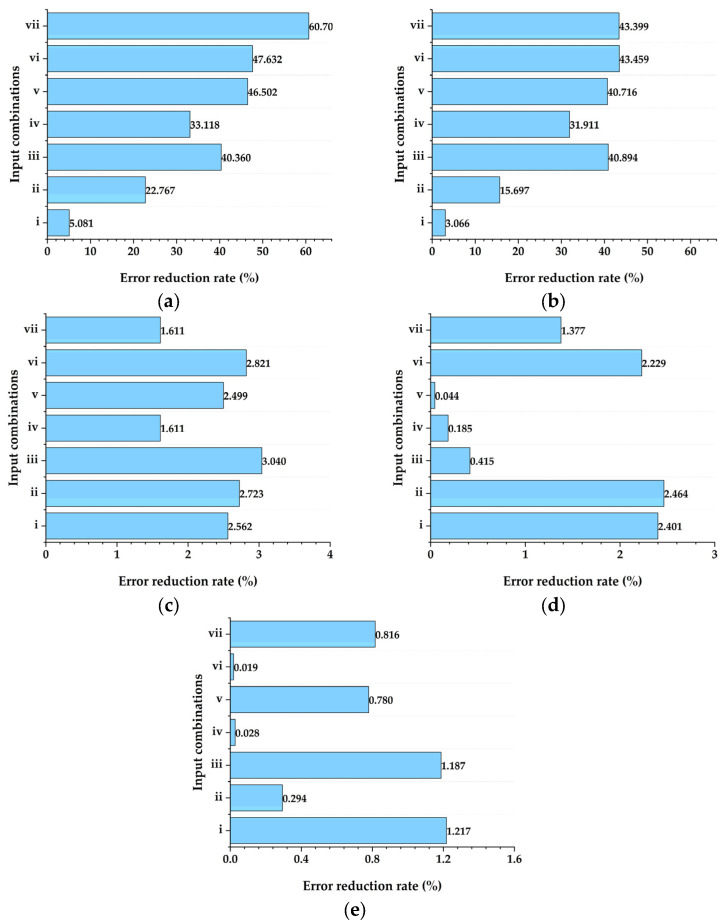
Error reduction rates (%) for various input combinations using the following algorithms: (**a**) PSO, (**b**) ACO, (**c**) GA, (**d**) DE, and (**e**) the GSA.

The reduction rates of the *MAE* (%) for different input combinations when metaheuristic algorithms (PSO, ACO, GA, DE and the GSA) are compared with the stand-alone RF algorithm are presented in Figure 4a–e. In particular, the RF-PSO model demonstrated a significant improvement, reducing the *MAE* by 5.081% to 60.707%, indicating its superior prediction accuracy and efficiency. Similarly, the RF-ACO model also showed a significant *MAE* reduction, ranging from 3.066% to 43.399% depending on the input combinations used. In contrast, the influence of the other models (RF-GA, RF-DE and RF-GSA) on *MAE* reduction was minimal or negligible, highlighting the limited effectiveness of these algorithms compared to RF-PSO and RF-ACO.

**Figure 5:**
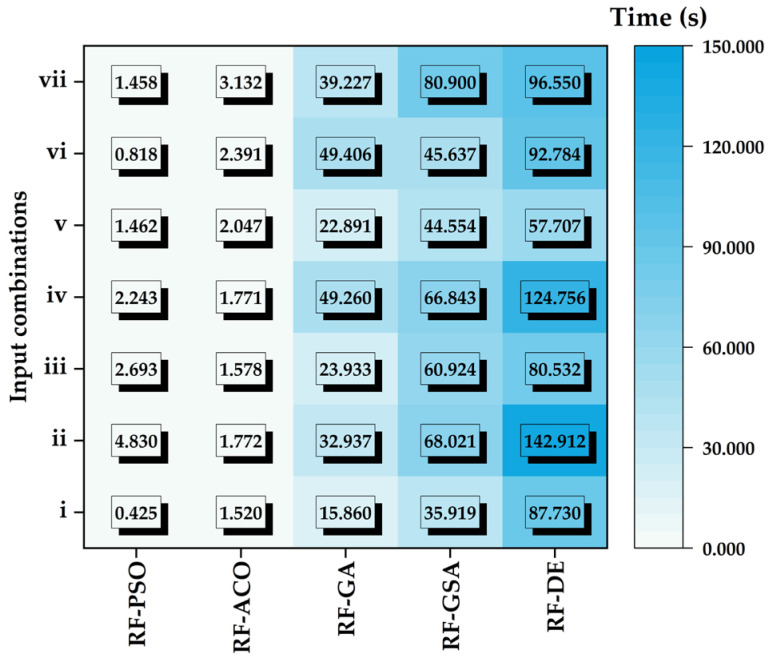
Computational efficiency comparison of different models based on the training time for each input combination.

Figure 5 shows the computational time (s) required to train metaheuristic algorithms (PSO, ACO, GA, DE and the GSA) for each input combination. In our study, we found that the time required to train RF models with PSO (0.425–4.80 s) and ACO (1.520–3.132 s) is quite similar, indicating their computational efficiency. In comparison, GA (15.860–49.406 s) exhibited moderate training times. However, the slower convergence speeds of the GSA (35.919–80.900 s) and DE (57.707–142.912 s) represented a notable limitation regarding these methods (Figure 5). Similar outcomes have been reported in various other studies, reinforcing the findings of this research [64,65].

PSO outperformed the other metaheuristic algorithms due to its strong capability in solving continuous problems [66]. The results of this study align with findings from other reports [67,68]. The PSO is characterized by its simple algorithmic design, which significantly increases its effectiveness in optimizing complex problems, such as tuning the hyperparameters of RF models. Thanks to this simplicity, PSO achieves fast convergence to optimal solutions while effectively balancing the exploration of new opportunities and the utilization of known successful regions in the search space. Therefore, PSO is particularly well suited for applications that require fast and reliable results.

Overall, the hybrid RF model is a viable solution for increasing operational efficiency in poultry farms. Its practical applications in weight forecasting, growth monitoring and feed optimization can lead to significant productivity gains and cost savings. Despite the initial cost and infrastructure requirements, the potential benefits and scalability of this solution make it suitable for farms of any size, even those with limited resources. With careful planning and support, the introduction of such models can contribute to more sustainable and profitable poultry farming.

Furthermore, future research to optimize these models and integrate them with new technologies will improve their applicability and effectiveness and ensure that the poultry industry is well equipped to meet future challenges and demands.

## 5. Conclusions

In this study, several metaheuristic algorithms, including PSO, ACO, GA, the GSA, and DE, were applied to train the RF model for predicting CW. The effectiveness of these algorithms in predicting CW was evaluated by generating various input combinations based on data collected from poultry houses, including T, RH, and FC. By systematically varying these inputs, the performance of the algorithms was rigorously assessed, facilitating the identification of optimal input combinations that significantly enhance their predictive capabilities. The key findings of this research are outlined below.

The combination of T, RH, and FC has been found to yield the most reliable estimates. However, it is also important to consider scenarios in cases where only climatic data are available, and the combinations of T and RH can also be used reliably.

Among the selected ML algorithms, the RF-PSO model, which incorporates T, RH, and F, achieved the best performance, with a *MAE* of 15.162 g and an *R* of 0.998. Additionally, the RF-ACO model, with the same inputs, demonstrated strong performance as well, with a *MAE* of 21.841 and an *R* of 0.997. In contrast, the influence of the other models (RF-GA, RF-DE, and RF-GSA) on the estimation was minimal or negligible, highlighting the limited effectiveness of these algorithms.

Furthermore, the computational time required to train the RF models using PSO and ACO was considerably low, indicating a high computational efficiency. In contrast, the GA exhibited moderate training times. Notably, the slower convergence speeds associated with the GSA and DE methods pose significant limitations in terms of their application.

In summary, the PSO and ACO algorithms have proven to be highly effective in improving the predictive performance of the RF model for estimating CW. The results show that these metaheuristic algorithms not only improve accuracy but also maintain computational efficiency, which makes them suitable for practical applications in poultry farming. Furthermore, the flexibility of these models in terms of providing reliable estimates with different input combinations, including scenarios with limited climatic data, emphasizes their versatility. Future research should focus on integrating additional variables, such as health and growth metrics, and exploring the real-time applicability of these models to further increase their utility in the poultry industry.

## Figures and Tables

**Table 1:** Descriptive statistics of the inputs and output used to train and test the models.

	Parameters	Unit	Min	Max	Mean	SD	Sk	Kr
Training	T	°C	20.030	30.550	25.649	2.997	−0.270	−0.695
	RH	%	56.410	72.780	64.178	3.865	0.054	−0.422
	FC	g	150.000	4448.000	1938.622	1470.146	0.429	−1.155
	CW	g	145.000	2714.000	1289.439	847.932	0.202	−1.311
Testing	T	°C	20.180	30.520	26.138	2.926	−0.393	−0.417
	RH	%	57.150	72.470	63.859	3.978	0.107	−0.519
	FC	g	158.000	4440.000	1863.362	1543.647	0.517	−1.188
	CW	g	157.000	2685.000	1238.448	895.216	0.326	−1.320

T: temperature, RH: relative humidity, FC: feed consumption, CW: chicken weight, Min: minimum, Max: maximum, Mean: Mean, SD: standard deviation, Sk: skewness, Kr: kurtosis.

**Table 2:** Performance metrics of RF and LR models for CW estimation.

Model	Inputs	Training		Testing	
		*MAE*	*R*	*MAE*	*R*
RF	T	216.097	0.906	291.134	0.855
	RH	340.817	0.806	394.552	0.767
	FC	26.520	0.999	39.639	0.997
	T and RH	75.406	0.983	112.374	0.958
	T and FC	27.631	0.999	37.755	0.998
	RH and FC	26.513	0.999	38.867	0.998
	T, RH, and FC	23.060	0.999	38.587	0.998
LR	T	345.192	0.831	387.818	0.792
	RH	648.554	0.380	664.290	0.502
	FC	82.816	0.993	80.043	0.994
	T and RH	331.533	0.856	376.514	0.823
	T and FC	72.837	0.994	73.812	0.995
	RH and FC	81.062	0.994	75.328	0.995
	T, RH, and FC	67.876	0.995	67.019	0.995

RF: Random Forest, LR: linear regression, T: temperature, RH: relative humidity, FC: feed consumption, CW: chicken weight, *MAE*: Mean Absolute Error, *R*: Correlation Coefficient.

**Table 3:** Configuration of the optimization algorithms employed in the study.

Algorithms	Parameters	i	ii	iii	iv	v	vi	vii
PSO	Cognitive component	1	1	2	1	1.5	1	2
	Social component	1.5	1.5	1	1	2	2	1.5
	Inertia weight	0.4	0.4	0.4	0.4	0.9	0.4	0.6
GA	Crossover rate	0.9	0.9	0.5	0.5	0.5	0.7	0.9
	Mutation rate	0.01	0.05	0.01	0.01	0.05	0.05	0.05
DE	Crossover probability	0.5	0.9	0.9	0.5	0.9	0.5	0.9
	Scaling factor	0.5	0.5	0.5	0.5	0.5	0.5	0.5
ACO	Pheromone evaporation rate	0.1	0.4	0.1	0.1	0.4	0.2	0.1
	Selection pressure	0.5	1	0.5	0.5	1.5	1	1.5
	Zeta	0.5	0.1	0.5	0.1	0.5	0.5	1
GSA	The coefficient of decrease	50	100	100	150	100	100	150
	The initial value of the gravitational constant	20	30	30	20	10	30	10
All algorithms	Population	30	30	30	30	30	30	30
	Number of iterations	200	200	200	200	200	200	200

Particle Swarm Optimization: PSO, Genetic Algorithm: GA, Ant Colony Optimization: ACO, Differential Evolution: DE, Gravity Search Algorithm: GSA.

**Table 4:** The comparison of different RF-based models in CW estimation.

Algorithms	Training		Testing	
	*MAE*	*R*	*MAE*	*R*
RF-PSO	7.138	0.999	15.162	0.998
RF-ACO	23.625	0.999	21.841	0.997
RF-GA	23.664	0.999	37.965	0.997
RF-GSA	23.815	0.999	38.272	0.997
RF-DE	22.773	0.999	38.056	0.997

Random Forest-Particle Swarm Optimization: RF-PSO, Random Forest-Ant Colony Optimization: RF-ACO, Random Forest-Genetic Algorithm: RF-GA, Random Forest-Gravity Search Algorithm: RF-GSA, Random Forest-Differential Evolution: RF-DE, *MAE*: Mean Absolute Error, *R*: Correlation Coefficient.

## Data Availability

The data presented in this study are available on request from the corresponding author.
